# The glycolytic enzyme PKM2 regulates inflammatory osteoclastogenesis by modulating STAT3 phosphorylation

**DOI:** 10.1016/j.jbc.2025.108389

**Published:** 2025-03-06

**Authors:** Mingjuan Li, Feng Li, Chongjie Zhu, Chi Zhang, Yushi Le, Zubing Li, Qilong Wan

**Affiliations:** State Key Laboratory of Oral & Maxillofacial Reconstruction and Regeneration, Key Laboratory of Oral Biomedicine Ministry of Education, Hubei Key Laboratory of Stomatology, School & Hospital of Stomatology, Wuhan University, Wuhan, China

**Keywords:** glycolysis, inflammation, M2 type pyruvate kinase, osteoclasts, periodontitis, p-STAT3

## Abstract

Periodontitis is a prevalent chronic inflammatory disease characterized by alveolar bone resorption mediated by osteoclasts. Pyruvate kinase M2 (PKM2), a key enzyme in glycolysis and pyruvate metabolism, has recently been recognized for its regulatory roles beyond metabolism, including gene expression and protein kinase activity. However, its exact role in osteoclastogenesis remains unclear. This study investigates the function of PKM2 in inflammatory osteoclastogenesis and explores its potential as a therapeutic target for periodontitis. Using murine bone marrow-derived macrophages (BMMs) stimulated with lipopolysaccharides (LPS) to mimic inflammatory conditions *in vitro*, we analyzed PKM2 expression and glycolytic activity during osteoclastogenesis through bioinformatics, tartrate-resistant acid phosphatase (TRAP) staining, phalloidin staining, quantitative real-time PCR (RT-qPCR), and Western blotting. Glycolysis was inhibited using 2-deoxy-D-glucose (2-DG), while TEPP-46 was used to activate PKM2. In a mouse model of periodontitis, the effects of TEPP-46 on alveolar bone loss were evaluated using micro-computed tomography, immunohistochemistry, TRAP staining, and hematoxylin-eosin (HE) staining. The results demonstrated that LPS significantly enhanced osteoclastogenesis and glycolysis, increasing PKM2 expression in osteoclasts. Inhibiting glycolysis with 2-DG suppressed osteoclast formation and osteoclast-related gene expression under inflammatory conditions. TEPP-46 treatment reduced nuclear dimeric PKM2 levels, decreased phosphorylated signal transducer and activator of transcription three (p-STAT3) expression, and inhibited osteoclastogenesis and osteoclast-related gene expression. Co-immunoprecipitation confirmed an interaction between nuclear dimeric PKM2 and p-STAT3. *In vivo*, TEPP-46 effectively reduced alveolar bone loss by preventing PKM2 nuclear translocation and STAT3 phosphorylation. These findings reveal that PKM2 regulates inflammatory osteoclastogenesis through modulation of glycolysis and STAT3 signaling, highlighting its potential as a therapeutic target for periodontitis.

Periodontitis is a common chronic inflammatory disease in the oral cavity, with alveolar bone resorption being its main issue (1, 2). Osteoclasts are multinucleated giant cells known to be the only cells in the skeletal system capable of bone resorption (3). Inflammation often leads to abnormalities in the quantity and function of osteoclasts, resulting in excessive bone resorption (4, 5, 6, 7). The formation of osteoclasts requires a high level of energy metabolism. Glucose is the primary energy source for osteoclasts and plays a crucial role in the skeletal system (8). Glycolysis is increased during osteoclast differentiation (9). When compared to the Bone Marrow-derived macrophages (BMM) group, bioinformatics analysis revealed a significant enhancement in glycolysis and pyruvate metabolism pathways in the osteoclast group (2). Sarah Rashid *et al.*'s research demonstrated that glycolytic genes were significantly upregulated in human osteoclast-like cells differentiated from peripheral blood mononuclear cells (PBMCs), as analyzed using RNA sequencing (10).

Inhibition of glycolysis inhibits osteoclastogenesis. Using inhibitors to block glycolysis or remove glucose from the culture medium has been shown to suppress osteoclast formation, indicating that glycolysis plays a key role in osteoclast differentiation (11, 12). Downregulation of glycolysis by reducing the production of lactate inhibits osteoclast formation (8). Glycolysis is the metabolic pathway in which glucose is metabolized into pyruvate in the cytoplasm, serving as a source of nutrients (13, 14). Pyruvate kinase (PK) is one of the key rate-limiting enzymes in glycolysis. In mammals, PK exists in four isoforms, but osteoclasts predominantly express pyruvate kinase M2 (PKM2), generated through alternative splicing of the PKM gene (15, 16, 17).

PKM2 exists in two main forms: a tetramer and a dimer. In its tetrameric form in the cytoplasm, PKM2 exhibits high glycolytic enzyme activity, efficiently converting phosphoenolpyruvate (PEP) to pyruvate (18). This pyruvate can then supply the tricarboxylic acid (TCA) cycle and oxidative phosphorylation (OXPHOS) or converted to lactate for secretion (19, 20). PKM2 mainly exists as a dimer in the nucleus. The glycolytic enzyme activity of dimeric PKM2 is lower than that of its tetrameric form (21). However, the expression of low-activity PKM2 is essential in proliferating cells, allowing for the accumulation of glycolytic intermediates necessary for pathways like nucleotide synthesis, such as the pentose phosphate pathway (PPP) (22). Some findings reveal that dimeric PKM2 has functions beyond its role as a glycolytic enzyme, such as regulating gene expression and protein kinase activity (23). Specifically, dimeric PKM2 translocates to the nucleus in cancer cells to stabilize the transcription factor hypoxia-inducible factor 1-alpha (HIF-1α) (24). Dimeric PKM2 also plays a crucial role in the activation of inflammatory macrophages (25). However, the importance of PKM2 in osteoclast formation remains largely unknown. A recent study suggested that phosphorylated PKM2 plays a role in osteoclast activation and function, reporting that inhibiting PKM2 activity can suppress osteoclast differentiation and prevent pathological bone loss (26). Thus, PKM2 activity may be essential for osteoclastogenesis, acting as a metabolic switch.

Up to now, there have been no studies investigating the effects of different forms of PKM2 on the excessive formation of osteoclasts under inflammatory conditions. In particular, research on the role of the dimeric form of PKM2 in the excessive formation of osteoclasts during inflammation remains virtually nonexistent. This study aims to elucidate the role of PKM2 in osteoclastogenesis under inflammatory conditions and to investigate the therapeutic potential of targeting PKM2 for periodontitis treatment.

## Result

### Inflammation significantly promotes osteoclast formation

**Figure 1:**
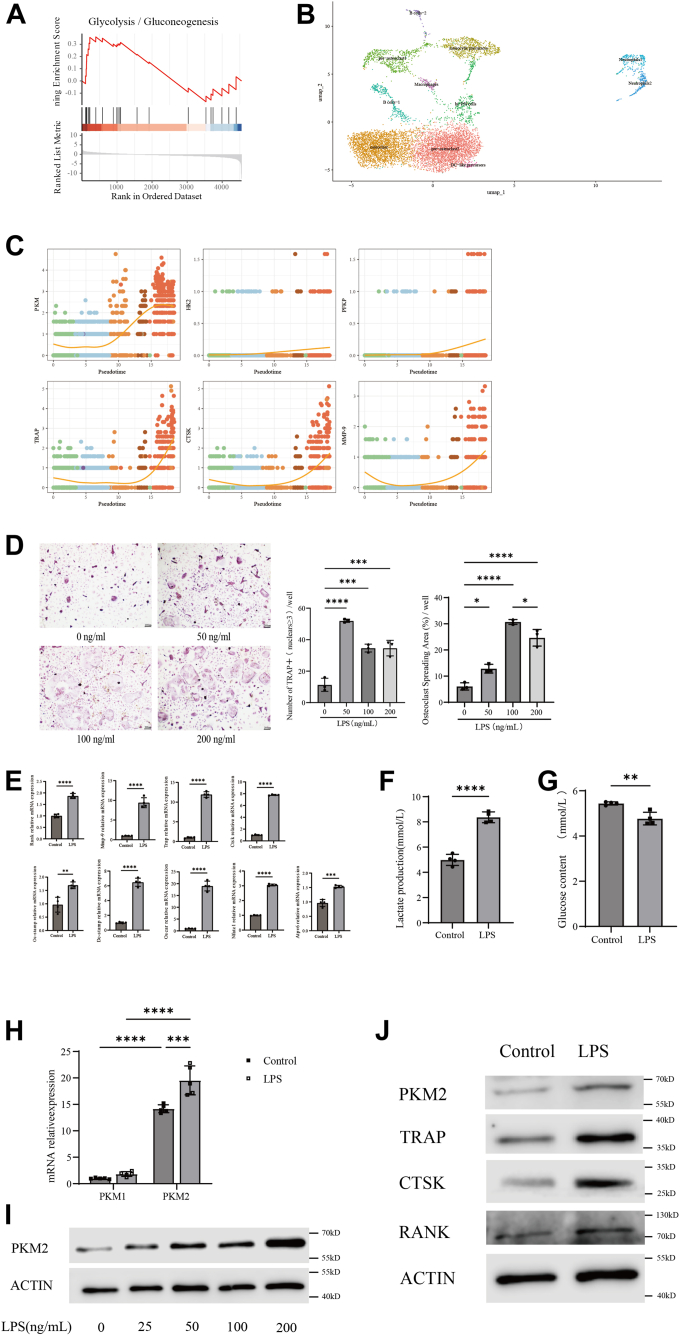
**Inflammation significantly promotes osteoclast formation.***A*, gene set enrichment analysis (GSEA) results indicate a significant difference in Glycolysis, comparing Bmms *versus* Osteoclast. *B*, definition of the clusters present in the osteoclast culture system on a UMAP visualization. *C*, the Gene expression of Key rate limiting enzymes in glycolysis and marker gene of osteoclast based on the Pseudotime. *D*, osteoclast precursors were co-cultured with different concentrations of lipopolysaccharides (LPS). n = 3 per group. Scale bars = 200 μm. *E*, quantitative real-time PCR analysis demonstrated that co-culture with LPS significantly upregulated the transcription of relevant proteins in osteoclasts. n = 4 per group. *F*, enzyme-linked immunosorbent assay (ELISA) analysis demonstrated that co-culture with LPS increased the production of lactic acid in osteoclasts. n = 4 per group. *G*, enzyme-linked immunosorbent assay (ELISA) analysis demonstrated that co-culture with LPS increased the consumption of glucose in osteoclasts. n = 4 per group. *H*, quantitative real-time PCR analysis demonstrated that the transcription level of PKM1 is significantly lower than that of PKM2, and stimulation with lipopolysaccharide markedly increases PKM2 transcription in osteoclasts. n = 5 per group. *I*, Western blot analysis revealed that expression of PKM2 proteins was effectively enhanced when co-culture with LPS in osteoclasts and exhibited a dose-dependent increase with the LPS concentration. *J*, Western blot analysis revealed that co-culture with LPS effectively enhanced the expression of TRAP, CTSK, PKM2 and RANK proteins in osteoclasts. Statistical analyses included unpaired Student’s *t* test or one-way ANOVA where appropriate. Data are presented as mean ± SD. ∗*p* < 0.05, ∗∗*p* < 0.01, ∗∗∗*p* < 0.001, ∗∗∗∗*p* < 0.0001. NS indicates not significant.

Gene Set Enrichment Analysis of the osteoclast formation process after filtration, standardization, and quality control revealed a significant enhancement of glycolysis (Fig. 1*A*). Glycolysis involves three key enzymes, namely pyruvate kinase M1/2 (PKM), hexokinase2 (HK2), and phosphofructokinase-1(PFK-1). Pseudotime analysis showed that during the induction of BMMs into osteoclasts, the expression of PKM gradually increased. The trend of increased PKM expression was consistent with the trend of osteoclast formation marker genes, such as TRAP, CTSK, and MMP-9. However, the expression levels of HK2 and PFK-1 did not show significant changes (Fig. 1, *B* and *C*). This suggests that PKM may play a crucial role in osteoclast formation. During inducing differentiation of murine BMMs into osteoclasts, different concentrations of LPS were added to the culture medium to simulate inflammation condition, with the 0 ng/ml group serving as the negative control group. After 5 days of culture, TRAP staining was performed on each group of cells. It was observed that with increasing LPS concentrations, there was an increase in the number of osteoclasts formed, as well as a significant enlargement in the volume of osteoclasts and an increased spread area of TRAP-positive osteoclasts (Fig. 1*D*). Following inflammatory stimulation, the expression of osteoclast-related genes such as *Rank*, *Mmp-9*, *Ctsk*, *Trap*, and *Oc-stamp* significantly increased. (Fig. 1*E*). This suggests that under inflammatory conditions, osteoclast formation is significantly enhanced. We examined two indicators of glycolysis and found that compared to the control group, the inflammatory stimulation group exhibited a significant increase in glucose consumption and lactate production, indicating enhanced glycolysis in osteoclasts under inflammatory conditions (Fig. 1, *F* and *G*). After the addition of LPS, PKM2 expression increased significantly, while PKM1 expression remained essentially unchanged. Moreover, PKM2 expression was consistently higher than PKM1 under both inflammatory and non-inflammatory conditions, indicating that PKM2 is the predominant isoform of pyruvate kinase involved in osteoclasts. It is noteworthy that following gradient inflammatory stimulation, the expression of PKM2 in osteoclasts significantly increased in a dose-dependent manner. (Fig. 1, *H* and *I*). Simultaneously, under inflammatory conditions, the protein expression of PKM2 significantly increased, consistent with the elevated levels of other osteoclast-related proteins such as TRAP, CTSK, and RANK(Fig. 1*J*). Our results indicate that inflammatory stimulation promotes osteoclast formation and significantly increases PKM2 expression, while also enhancing glycolysis.

### 2-DG effectively reduces the promotion of osteoclastogenesis induced by inflammation

**Figure 2:**
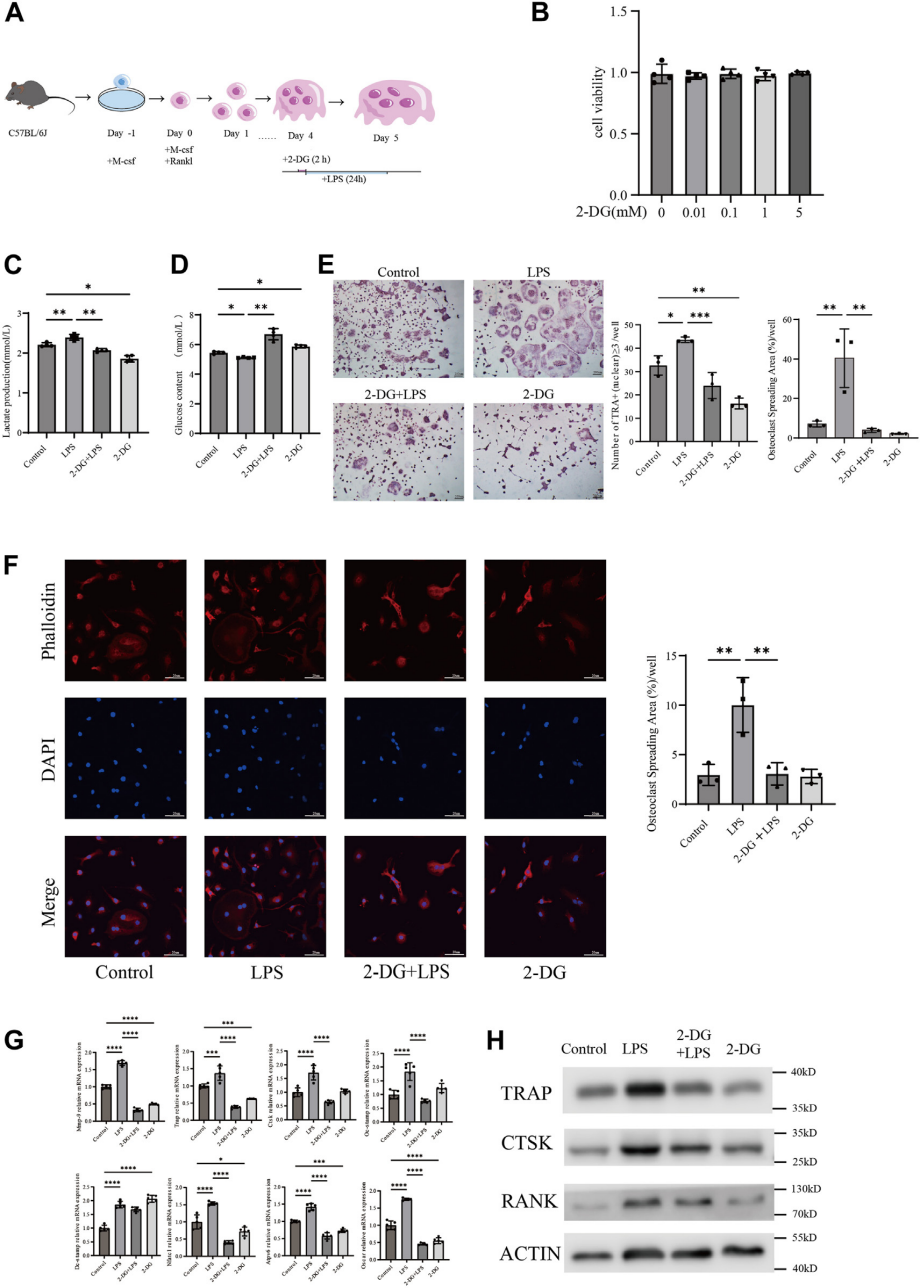
**2-DG effectively reduces the promotion of osteoclastogenesis induced by inflammation.***A*, schematic diagram of osteoclastogenesis from the bone marrow-derived macrophages of C57BL/6J mice; supplementation of 2-dg (5 mM) was done on the fourth day for 2 h, then with or without stimulation from day 4 to day 5. *B*, effect of 2-dg supplementation on cell viability in BMMs for 2 h on the day 5. n = 4 per group. *C*, enzyme-linked immunosorbent assay (ELISA) analysis showed that co-culture with LPS increased lactic acid production in osteoclasts, while the addition of 2-DG reduced lactic acid production. n = 4 per group. *D*, ELISA analysis demonstrated that co-culture with LPS increased glucose consumption in osteoclasts, while the addition of 2-DG reduced glucose consumption. n = 4 per group. *E*, osteoclast precursors were co-cultured with or without LPS, with or without 2-dg according to experiments’ protocol. TRAP staining results indicated that 2-dg exhibited greater efficacy in suppressing osteoclast overactivation in inflammatory conditions. n = 3 per group. Scale bars = 200 μm. *F*, immunofluorescence staining with phalloidin for F-actin and DAPI for the nucleus was performed to assess the effect of co-culture with or without LPS and 2-dg on osteoclast size. As the supplementation of LPS, a noticeable increase in the size of osteoclasts was observed. while supplementation of 2-dg can restrain this trend. The osteoclast spreading area (%)/well refers to the proportion of the fluorescent area of osteoclasts stained with phalloidin relative to the total field of view. n = 3 per group. Scale bars = 20 μm. *G*, quantitative real-time PCR analysis demonstrated that co-culture with LPS significantly upregulated the transcription of relevant proteins in osteoclasts. n = 5 per group. *H*, Western blot analysis revealed that co-culture with LPS effectively enhanced the expression of TRAP, CTSK and RANK proteins in osteoclasts. One-way ANOVA was used. Data are presented as mean ± SD. ∗*p* < 0.05, ∗∗*p* < 0.01, ∗∗∗*p* < 0.001, ∗∗∗∗*p* < 0.0001. NS indicates not significant.

To further investigate the role of glycolysis in osteoclast formation under LPS stimulation, we selected the glycolysis inhibitor 2-DG for further exploration and a specific experimental procedure is shown in the figure below (Fig. 2*A*). Initially, 2-DG treatment (5 mM for 2 h) is not cytotoxic to BMMs (Fig. 2*B*). After the addition of 2-DG, the measurement of lactate production and glucose levels, both indicators of glycolysis, showed that glycolysis in osteoclasts was inhibited, regardless of LPS stimulation (Fig. 2, *C* and *D*). From the TRAP staining images, it is evident that, even under inflammatory stimulation, osteoclastogenesis is significantly reduced following 2-DG treatment (Fig. 2*E*). With the supplementation of 2-DG, a noticeable decrease in the size of osteoclasts was observed (Fig. 2*F*). Two-DG markedly suppresses the transcriptional levels of genes associated with osteoclastogenesis under inflammatory conditions (Fig. 2*G*). Additionally, the expression of osteoclast-related proteins TRAP, CTSK, and RANK is also notably decreased (Fig. 2*H*). These findings indicate that inhibition of glycolysis following inflammatory stimulation significantly suppresses osteoclastogenesis.

### TEPP-46 significantly reduces the promotion of osteoclastogenesis induced by inflammation

**Figure 3:**
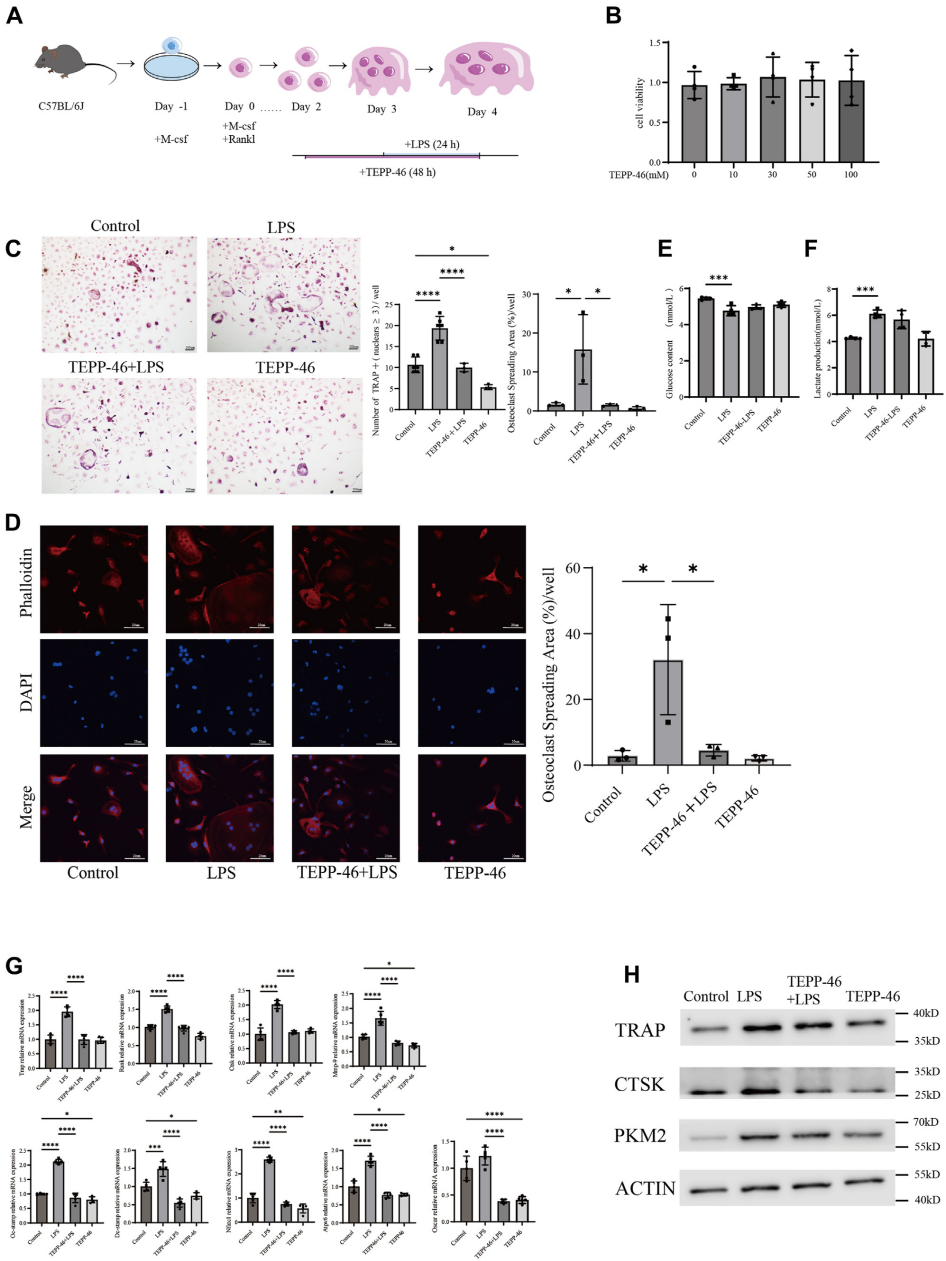
**TEPP-46 significantly reduces the promotion of osteoclastogenesis induced by inflammation.***A*, schematic diagram of osteoclastogenesis from the bone marrow-derived macrophages of C57BL/6J mice; supplementation of TEPP-46 (5 mM) was done from day 2 to day 4, then with or without stimulation with 100 ng/ml LPS from day 3 to day 4. *B*, effect of TEPP-46 supplementation on cell viability in BMMs from day 2 to day 4. n = 4 per group. *C*, osteoclast precursors were co-cultured with or without LPS, with or without TEPP-46 according to experiments’ protocol. TRAP staining results indicated that TEPP-46 exhibited greater efficacy in suppressing osteoclast overactivation in inflammatory conditions. n = 3 per group. Scale bars = 200 μm. *D*, immunofluorescence staining with phalloidin for F-actin and DAPI for the nucleus was performed to assess the effect of co-culture with or without LPS and TEPP-46 on osteoclast size. As the supplementation of LPS, a noticeable increase in the size of osteoclasts was observed. while supplementation of TEPP-46 can restrain this trend. The osteoclast spreading area (%)/well refers to the proportion of the fluorescent area of osteoclasts stained with phalloidin relative to the total field of view. n = 3 per group. Scale bars = 20 μm. *E*, ELISA analysis demonstrated that co-culture with LPS increased the production of lactic acid in osteoclasts, the addition of TEPP-46 has no effect on lactate production. n = 4 per group. *F*, ELISA analysis demonstrated that co-culture with LPS increased the consumption of glucose in osteoclasts, the addition of TEPP-46 has no effect on l the consumption of glucose. n = 4 per group. *G*, quantitative real-time PCR analysis demonstrated that co-culture with LPS significantly upregulated the transcription of relevant proteins in osteoclasts, while supplementation of TEPP-46 can restrain this trend. n = 5 per group. *H*, Western blot analysis revealed that co-culture with LPS effectively enhanced the expression of TRAP, CTSK and PKM2 proteins in osteoclasts, while supplementation of TEPP-46 can restrain this trend. n = 3 per group. One-way ANOVA was used. Data are presented as mean ± SD. ∗*p* < 0.05, ∗∗*p* < 0.01, ∗∗∗*p* < 0.001, ∗∗∗∗*p* < 0.0001. NS indicates not significant.

Given that inhibition of glycolysis suppresses osteoclastogenesis, we further investigated whether activation of one of the key rate-limiting enzymes in glycolysis, PKM2, could promote osteoclastogenesis. We subsequently utilized the PKM2 activator TEPP-46 to examine whether increased expression of PKM2 could enhance osteoclastogenesis and a specific experimental procedure is shown in the figure below (Fig. 3*A*). Initially, TEPP-46 treatment (50 μM for 48 h) is not cytotoxic to BMMs (Fig. 3*B*). However, TRAP staining revealed that the addition of TEPP-46 did not promote osteoclastogenesis under either inflammatory or non-inflammatory conditions (Fig. 3*C*). With the supplementation of TEPP-46, a noticeable decrease in the size of osteoclasts was observed with or without LPS (Fig. 3*D*). TEPP-46 does not affect glycolysis in osteoclasts (Fig. 3, *E* and *F*), while it also increases PKM2 protein expression (Fig. 3*H*). Moreover, transcriptional levels of osteoclast-related genes and protein expression were inhibited (Fig. 3, *G* and *H*). Our results demonstrate that while inhibition of osteoclast glycolysis with 2-DG significantly suppresses osteoclastogenesis, activation of PKM2 using TEPP-46 does not promote osteoclastogenesis. Therefore, the regulatory mechanism of PKM2 in osteoclast formation requires further investigation.

### Nuclear PKM2 regulates osteoclastogenesis through the phosphorylation of STAT3

**Figure 4:**
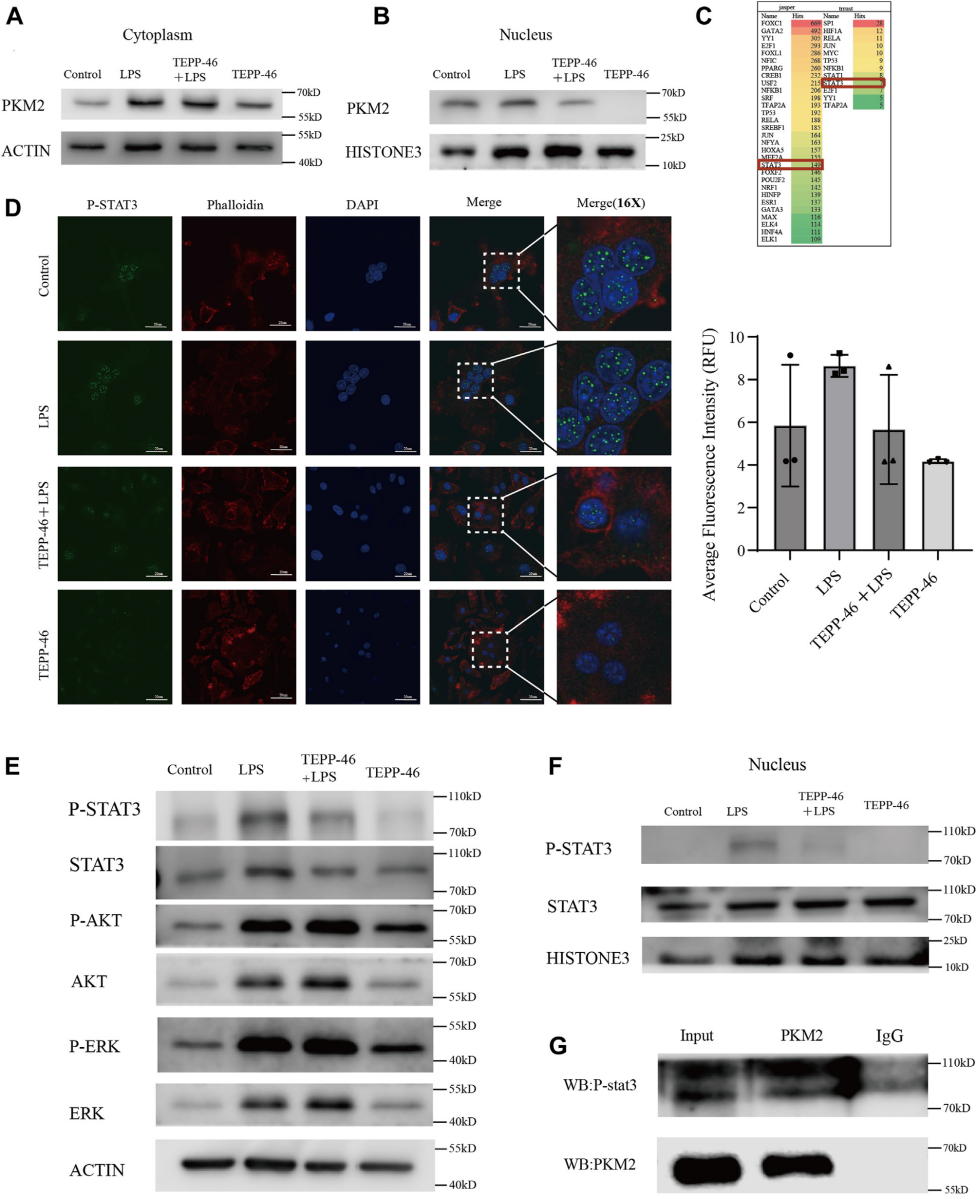
**Nuclear PKM2 regulates osteoclastogenesis through the phosphorylation of STAT3.***A*, cytoplasmic expression of PKM2 was assessed in Control, LPS, TEPP-46 + LPS, and TEPP-46 treated groups using a nuclear-cytoplasmic fractionation assay. *B*, nuclear expression of PKM2 was assessed in Control, LPS, TEPP-46 + LPS and TEPP-46 treated groups using a nuclear-cytoplasmic fractionation assay. TEPP-46 treatment resulted in a significant decrease in nuclear PKM2 expression. *C*, transcription factors enriched from two databased with the differentially expressed genes in RNA-seq data comparing Bmms *versus* Osteoclast. *D*, phalloidin was used to stain F-actin, DAPI to stain nuclei, and fluorescent secondary antibodies to stain p-STAT3 in osteoclasts. The results show that p-STAT3 expression in the LPS-stimulated group is significantly higher than in the control group. Scale bars = 20 μm. *E*, Western blot analysis of p-STAT3, STAT3, p-AKT, AKT, p-ERK, and ERK protein levels. TEPP-46 treatment significantly decreased p-STAT3 expression, while p-AKT and p-ERK levels remained unchanged. *F*, nuclear expression of p-STAT3 was assessed in Control, LPS, TEPP46-LPS and TEPP46 treated groups using a nuclear-cytoplasmic fractionation assay. TEPP-46 treatment resulted in a significant decrease in nuclear p-STAT3 expression. *G*, co-immunoprecipitation results indicate that p-STAT3 binds to PKM2 in osteoclasts.

Western blot analysis of PKM2 expression in the nucleus and cytoplasm of control and inflammation-stimulated groups revealed a significant increase in nuclear PKM2 in the inflammation-stimulated group. However, upon the addition of TEPP-46, nuclear PKM2 levels were significantly reduced (Fig. 4, *A* and *B*). This suggests that nuclear pyruvate kinase dimers play a crucial role in osteoclast formation.

To further investigate the role of PKM2 in osteoclast formation, we performed transcription factor enrichment analysis using sequencing data from relevant databases. STAT3 was found to be enriched in two transcription factor databases of differentially expressed genes (DEGs) (Fig. 4*C*). Simultaneously, immunofluorescence staining revealed that under LPS stimulation, the fluorescence intensity of p-STAT3 was enhanced, whereas the addition of TEPP-46 reversed this trend (Fig. 4*D*). Western blot revealed that the protein expression levels of p-STAT3 were significantly elevated in the inflammation-stimulated group, while the addition of TEPP-46 inhibited p-STAT3 expression. This trend was not observed with other protein factors (Fig. 4*E*). After extracting nuclear proteins, it was observed that inflammatory stimulation significantly increased p-STAT3 levels, whereas the addition of TEPP-46 reduced p-STAT3 levels (Fig. 4*F*). Subsequent co-immunoprecipitation experiments confirmed the interaction between pyruvate kinase and p-STAT3 (Fig. 4*G*). Our results demonstrate that nuclear PKM2 regulates osteoclastogenesis through the phosphorylation of STAT3.

### TEPP-46 has therapeutic effect on mouse periodontitis model

**Figure 5:**
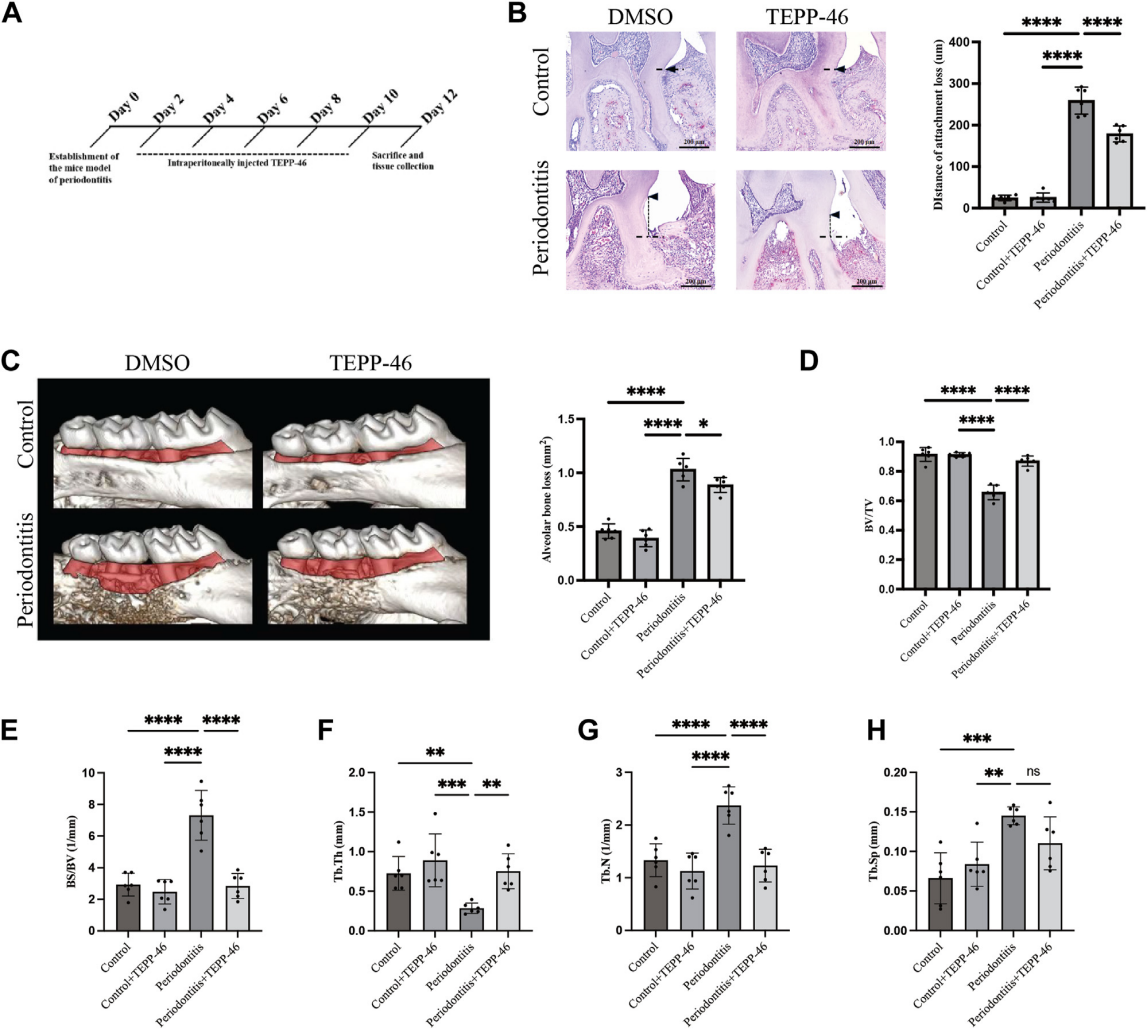
**TEPP-46 alleviates the loss of bone resorption and attachment induced by periodontitis.***A*, the experimental design schematic illustrates the preparation of the periodontitis model through silk ligation and the timeline for administration (n = 6 per group). *B*, HE staining of periodontal tissue was utilized to assess epithelial attachment loss. The *black* triangle indicates the enamel-dentin junction, the *black* horizontal dashed line denotes the *bottom* of the periodontal pocket, and the *black* vertical dashed line represents attachment loss (n = 6 per group). *C*, micro-CT scans and 3D reconstructions of periodontal tissue were conducted to evaluate bone loss. *D*–*H*, trabecular separation (Tb.Sp), bone volume/tissue volume ratio (BV/TV), bone surface/volume ratio (BS/BV), trabecular thickness (Tb.Th), and trabecular number (Tb.N) were employed to assess alveolar bone quality (n = 6 per group). Statistical analyses included unpaired Student’s *t* test or one-way ANOVA where appropriate. Data are presented as mean ± SD. ∗*p* < 0.05, ∗∗*p* < 0.01, ∗∗∗*p* < 0.001, ∗∗∗∗*p* < 0.0001. NS indicates not significant.

Based on *in vitro* experiments demonstrating that TEPP-46 reduces osteoclast formation in an inflammatory environment by inhibiting PKM2 nuclear translocation, we further investigated the potential of TEPP-46 in treating periodontitis. The mouse model of periodontitis was established using molar silk ligation, followed by intraperitoneal administration of TEPP-46 every other day, as referenced in a previous study (Fig. 5*A*). The experimental results indicated that, compared with normal mice without ligature, the mice with ligature exhibited significant loss of epithelial attachment and alveolar bone, confirming the successful establishment of the periodontitis model (Fig. 5, *B* and *C*). HE staining of the periodontal tissues in mice with periodontitis revealed that TEPP-46 treatment significantly mitigated the loss of epithelial attachment (Fig. 5*B*). Micro-CT scans and 3D reconstruction analyses showed that TEPP-46 significantly reduced bone loss associated with periodontitis (Fig. 5*C*). Although TEPP-46 did not completely prevent trabecular separation (Tb.Sp) in the alveolar bone of periodontitis mice (Fig. 5*H*), other indicators such as bone volume/tissue volume ratio (BV/TV), bone surface/volume ratio (BS/BV), trabecular thickness (Tb.Th), and trabecular number (Tb.N) were significantly improved (Fig. 5, *D*–*G*). Therefore, these results support the conclusion that TEPP-46 is effective in treating both soft and hard tissue injuries caused by periodontitis.

### TEPP-46 inhibits osteoclastogenesis by preventing PKM2 nuclear translocation, thereby weakening STAT3 phosphorylation

**Figure 6:**
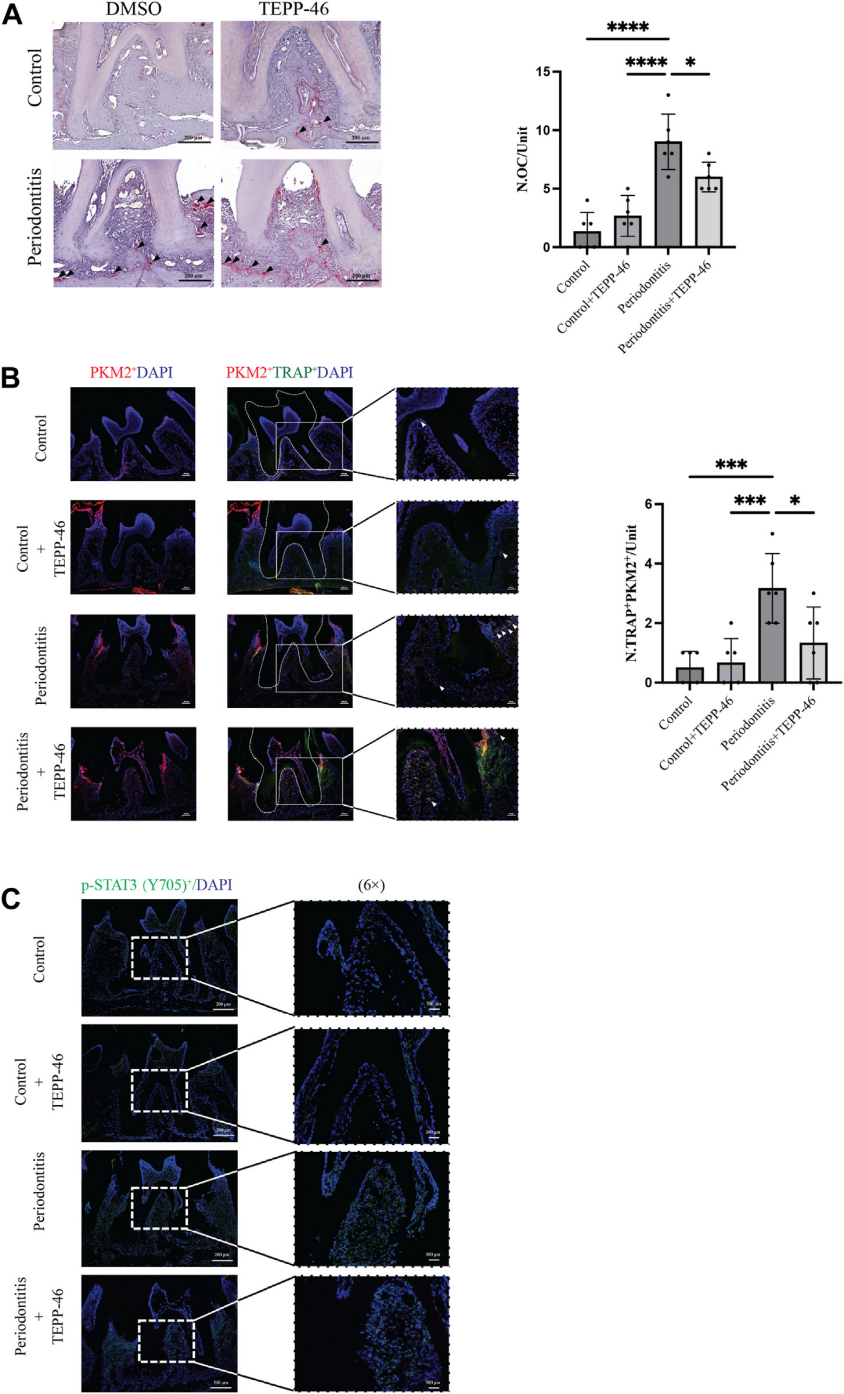
**TEPP-46 inhibits osteoclast formation by reducing STAT3 phosphorylation levels.***A*, TRAP staining was conducted on periodontal tissues to assess TEPP-46's impact on osteoclast generation in alveolar bone. The *black* triangle indicates TRAP-positive cells (n = 6 per group). *B*, representative images of PKM2 (*red*), TRAP (*green*), and DAPI (*blue*) immunofluorescence co-staining in periodontal tissue. TEPP-46's effect on PKM2 activation was evaluated, and alveolar bone tissues around the second M were locally enlarged to detect osteoclasts with positive PKM2 expression (PKM2/TRAP double-positive cells) (n = 6 per group). *C*, to evaluate TEPP-46's impact on STAT3 phosphorylation (*green*) in periodontal tissue, p-STAT3 was stained *via* immunofluorescence and restained with DAPI (blue) (n = 6 per group). Local magnification of the alveolar bone space of the second M was performed to assess STAT3 phosphorylation levels. Statistical analyses included unpaired Student’s *t* test or one-way ANOVA where appropriate. Data are presented as mean ± SD. ∗*p* < 0.05, ∗∗*p* < 0.01, ∗∗∗*p* < 0.001, ∗∗∗∗*p* < 0.0001. NS indicates not significant.

To further elucidate the mechanism of TEPP-46 in treating periodontitis, we performed TRAP staining on the alveolar bone tissue of mice. Osteoclasts were significantly increased in the periodontitis model compared to normal periodontal tissue. This increase in osteoclasts was directly linked to bone loss in periodontitis. TEPP-46 intervention significantly reduced the number of osteoclasts in periodontitis (Fig. 6*A*). We explored how TEPP-46 reduces osteoclasts *in vivo* by performing dual immunofluorescence staining of TRAP protein (red) and PKM2 protein (green) in periodontal tissue. TEPP-46, acting as a PKM2 agonist, increased the expression of PKM2 (red) in periodontitis tissues, while significantly inhibiting PKM2-positive osteoclasts (PKM2+TRAP+) (Fig. 6*B*). This suggests that TEPP-46 does not promote PKM2’s role in glycolysis, supporting *in vitro* findings. To verify PKM2's action as a protein kinase, we measured p-STAT3, a target protein of PKM2 kinase. The results showed that p-STAT3 levels in periodontal tissues significantly decreased after TEPP-46 inhibited PKM2 nuclear translocation (Fig. 6*C*). These *in vivo* results suggest that TEPP-46’s therapeutic effect on periodontitis comes from inhibiting PKM2 nuclear translocation, thereby reducing STAT3 phosphorylation and subsequently reducing osteoclastogenesis.

## Discussion

Increased alveolar bone resorption caused by osteoclast dysregulation or heightened osteoclastogenesis is a major mechanism underlying alveolar bone loss in periodontitis (3, 27). Currently, many treatments for periodontitis target osteoclasts (*e.g.*, estrogen replacement, bisphosphonates, and anti-RANKL antibodies), but these approaches are associated with adverse effects such as atypical fractures, delayed healing, heart attacks, and osteonecrosis of the jaw (28, 29, 30). Therefore, understanding the mechanisms that influence osteoclast formation remains of great importance. In our study, firstly, bioinformatics analysis revealed that PKM2 expression significantly increases during osteoclastogenesis and aligns with the expression trends of osteoclast marker genes (such as *Trap*, *Ctsk*, and *Mmp-9*). In our previous study, we found that silencing PKM2 using siPKM2 significantly reduced the mRNA transcription levels of *Ctsk*, *Trap*, and *Rank* as well as osteoclast formation. Additionally, under inflammatory stimuli, PKM2 protein expression increases in a dose-dependent manner, indicating a potential critical role of PKM2 in osteoclast formation. We had utilized shikonin, a PKM2 inhibitor, to investigate its effects. Shikonin was shown to inhibit osteoclastogenesis under inflammatory stimulation *in vitro* and reduce alveolar bone loss in a mouse model of periodontitis (31). Similarly, Cai *et al.* found that the supernatant of odontogenic keratocyst fibroblasts induces the formation of mature osteoclasts in RAW264.7 cells, while PKM2 knockdown attenuates this effect (32).

PKM2 exists mainly in two isoforms: a tetramer primarily located in the cytoplasm, which has high glycolytic enzyme activity, converting phosphoenolpyruvate and ADP to pyruvate and ATP; and a dimer mainly found in the nucleus, where it acts as a protein kinase phosphorylating various protein targets, contributing to various physiological and pathological processes (33, 34). For instance, PKM2-dependent phosphorylation of H3 facilitates the removal of histone deacetylase 3 (HDAC3) from the promoters of CCND1 and MYC. This process results in the acetylation of H3 at Lys9 and the EGF-triggered transcription of cyclin D1 and c-Myc, which in turn enhances cyclin D1-driven cell cycle progression and tumor formation (35). Phosphorylation of AKT1 substrate 1 (AKT1S1) at Ser202/203 by PKM2 facilitates its interaction with 14-3-3, triggering mTORC1 signaling and driving oncogenic growth in cancer cells (36). Through bioinformatics analysis, we discovered enhanced glycolysis during osteoclast formation compared to BMMs, with PKM2 being a key rate-limiting enzyme in glycolysis. We inhibit glycolysis using 2-DG, which significantly reduces osteoclast formation and the transcription of related genes, even under inflammatory stimuli, verifying the crucial role of glycolysis in osteoclastogenesis. This is consistent with the findings of Helen J. Knowles' study (37). Recent studies suggest that the energy produced through glycolysis also plays a significant role in osteoclast formation (8).

It is unclear whether the dimeric form of PKM2 in the nucleus plays a role in osteoclast formation. From the literature, we learned that the reagent TEPP-46 increases cytoplasmic tetrameric PKM2, reduces nuclear translocation of PKM2, and decreases nuclear dimeric PKM2 (38, 39). Through the application of Western blot analysis to both nuclear and cytoplasmic proteins derived from control and inflammatory-stimulated groups, we observed a significant increase in the nuclear dimer PKM2 within the inflammatory-stimulated group. Conversely, the introduction of TEPP-46 to the nucleus resulted in a notable decrease in the dimer PKM2.This suggests that nuclear dimeric PKM2 may play a key role in the increased osteoclast formation caused by inflammatory stimuli. To further explore PKM2's transcriptional regulation in osteoclastogenesis, we performed transcription factor enrichment analysis using sequencing data from relevant databases. Interestingly, STAT3 was enriched in two DEG transcription factor databases. Yang P’s research demonstrated that LPS enhances PKM2 binding to the STAT3 promoter, which in turn promotes STAT3 transcription and nuclear translocation, leading to the secretion of pro-inflammatory cytokines and increased cell proliferation in colorectal cancer. The nuclear translocation of STAT3 mediated by PKM2, along with the protein kinase activity of dimeric PKM2, is critical for colorectal cancer cell migration and adhesion. Additionally, the activation of STAT3 by nuclear PKM2 decreases the sensitivity of colorectal cancer cells to EGFR pathway tyrosine kinase inhibitors (40, 41).

The results of the Western blotting experiment demonstrated a significant increase in p-STAT3 protein expression within the inflammatory stimulation group. However, this trend was inhibited by TEPP-46. No such trend was observed for other protein factors. Co-immunoprecipitation confirmed the interaction between PKM2 and p-STAT3, suggesting that nuclear PKM2 might regulate osteoclast formation through interaction with p-STAT3. In this study, we report that the small molecule activator TEPP-46 induces the formation of tetrameric PKM2, blocks nuclear translocation and dimer formation of PKM2, inhibits osteoclast formation and bone resorption *in vitro*, and alleviates alveolar bone loss in experimental periodontitis in mice, reducing the severity of periodontitis. And nuclear PKM2 might regulate osteoclast formation through interaction with p-STAT3. PKM2 regulates inflammatory osteoclastogenesis by modulating both the glycolysis pathway and STAT3 phosphorylation levels. Our findings suggest that pharmacologically targeting PKM2 may be a valuable approach for treating osteoclast-related diseases such as periodontitis.

Based on our findings, it remains a challenge to investigate whether increasing nuclear dimeric PKM2 expression would enhance osteoclast formation and STAT3 phosphorylation. Future research should explore the interactions between PKM2 and other transcription factors and their specific regulatory mechanisms in osteoclast formation. Given the unique role of TEPP-46 in regulating PKM2 subcellular localization, developing more specific drugs to modulate PKM2 activity and localization could offer new therapeutic avenues for osteoclast-related diseases.

## Materials and methods

### Animals and ethics statement

C57BL/6J male mice, 6 weeks old, were procured from the Hubei Laboratory Animal Research Center. Following a 7-day acclimatization period, periodontitis was induced in the mice. The animals were housed under controlled environmental conditions, with a temperature of 22 ± 1 °C, humidity maintained at 55 ± 5%, and a 12-h light/dark cycle. Standard rodent chow and water were provided ad libitum throughout the study. All experimental procedures were conducted in compliance with the animal care guidelines established by Wuhan University and were approved by the Wuhan University Committee on Ethics in the Use of Laboratory Animals (Project Number: S07924020I).

### Data processing for gene expression and differential gene expression analysis

The original data from GSE147174 included single-cell RNA-seq data of an osteoclast culture system on days 0, 1, and 3 (2). Computational analyses were performed using the Seurat R package. Quality control was performed by selecting viable cells, excluding genes expressed in fewer than three cells, and cells expressing between 200 and 2500 genes with less than 20% mitochondrial reads. Read counts per cell were normalized and scaled. Principal component (PC) analysis was used for dimensionality reduction, and uniform manifold approximation and projection (UMAP) was performed on the first 20 PC dimensions. Cells were assigned to clusters using the Find Neighbors function in Seurat, and the UMAP clusters were identified using the Find Clusters function with a resolution of 0.5. Differential expression analysis was performed on the bulk RNA-seq data from GSE147174 using DESeq2. Gene Set Enrichment Analysis (GSEA) was conducted with the clusterProfiler R package based on the KEGG database. Transcription factors were enriched using Network Analyst 3.0. Pseudotime analysis was performed using the Slingshot package (version 2.10.0) with a threshold of 0.001, ending with the "osteoclast" cluster. Gene expression was visualized using the Seurat package and ggplot2.

### Osteoclast culture and quantification

BMMs were harvested from mouse femurs according to the protocol described in ‘Bone Research Protocols' (42). The procedure is outlined in the following steps, which provide a general overview. The femurs were dissected under sterile conditions, and bone marrow was flushed out with α-MEM (GIBCO, Invitrogen) using a 1 ml syringe. Collected cells were centrifuged and cultured in T-25 flasks with 10 ng/ml M−CSF (Novoprotein) for 24 h at 37 °C and 5% CO_2_. After 24 h, adherent cells identified as osteoclast precursors (OCPs) were harvested. OCPs were seeded onto plates at a concentration of 1 × 10^6^ cells/ml and cultured for 4 or 6 days with 20 ng/ml M−CSF and 50 ng/ml RANKL (Novoprotein), with medium changes every other day. After 6 days of culture, cells were fixed with 4% paraformaldehyde for 10 min and stained using the leukocyte acid phosphatase kit (Sigma Aldrich) according to the manufacturer's instructions. TRAP-positive multinucleated cells with more than three nuclei were quantified.

### Cell viability assay

OCPs were seeded in 96-well plates at a concentration of 1 × 10^4^ cells/well in the presence of 20 ng/ml M−CSF and 50 ng/ml RANKL, with medium changes every other day. TEPP-46 (Cat.# HY-18657) and 2-DG (Cat.# HY-13966) were acquired from MedChemExpress (Monmouth Junction). TEPP-46 was diluted with dimethyl sulfoxide (DMSO), while 2-DG was diluted with double-distilled water. TEPP-46 was added starting from the third day, and cell viability was assessed using the Cell Counting Kit-8 (CCK-8, Beyotime, Cat. C0037) after a 2-days incubation period. The experiment regarding the cell viability assay of 2-DG, on the fourth day, 2-DG was added and allowed to incubate for 2 h. Then on the fifth day, perform the CCK-8 assay. Absorbance (optical density, OD) was measured at 450 nm using an ELISA plate reader.

### DAPI and phalloidin staining

Cells were fixed with 4% paraformaldehyde for 10 min at 37 °C, followed by permeabilization using 0.5% Triton-100 for 5 min at room temperature. To label F-actin, a mixture of phalloidin-Alexa 488 and 1% bovine serum albumin in a 1:300 ratio was applied to the cells and incubated in the dark for 30 min. Subsequently, the cells were stained with DAPI for 10 min. Images were analyzed using ImageJ software. The osteoclast spreading area was determined as the ratio of the phalloidin-stained osteoclast area to the total area of the field of view.

### Quantitative real-time PCR

Total RNA was extracted from osteoclasts after 4 days of differentiation using the EZNA Total RNA Kit (Omega). Subsequently, cDNA was synthesized using the PrimeScript RT Reagent Kit (Takara). Quantitative real-time polymerase chain reaction (RT-qPCR) was conducted in triplicate using the SYBR Green Realtime PCR Master Mix (TOYOBO). Expression levels of target genes were normalized to β-actin, and relative gene expression levels were calculated using the 2−ΔΔCT method. The sequences of PCR primers were presented in Supplementary Table S1.

### Glucose uptake, L-lactate detection assay

OCPs were seeded at a density of 2 × 10^5^ cells per well in 24-well plates. The culture medium, with or without LPS or 2-DG, was collected for analysis of lactic acid and glucose levels using the lactic acid detection kit (Jiancheng) and glucose content kit (Jiancheng), respectively. Measurements were performed using a Bio-Rad 680 Microplate Reader, following the manufacturer's instructions. Standard curves were generated using lactate and glucose standard samples to calculate the absolute content of lactic acid and glucose. OCPs cultured with only M−CSF and RANKL served as the control group, while LPS and 2-DG were added to the experimental groups.

### Western blotting

Osteoclasts differentiated for 6 days were washed with PBS to remove extracellular proteins and intracellular proteins were extracted using RIPA buffer (Jiancheng). Proteins were separated using SDS-PAGE and transferred onto polyvinylidene fluoride (PVDF) membranes (Millipore). Membranes were incubated overnight at 4 °C with primary antibodies (diluted 1:1000): rabbit anti-TRAP (Abcam, Cat. # ab133238), rabbit anti-RANK (Abclonal, Cat. #A12997), rabbit anti-CTSK (Abclonal, Cat. #A1782), rabbit anti-PKM2 (Abclonal, Cat. #A22408), and mouse anti-β-actin (Abclonal, Cat. #AC004). PVDF membranes were then incubated with Goat Anti-Rabbit (Abclonal, Cat. #AS014) or Goat Anti-Mouse IgG H&L (HRP) (Abclonal, Cat. #AS003) secondary antibodies (diluted 1:5000) for 1 h at room temperature. Protein bands were visualized using HRP substrate luminol reagent (Millipore), and the chemiluminescent signal was detected using the Image Studio System (LI-COR, NE). Densitometric analysis was performed using ImageJ software, and protein quantification was achieved by determining the grey value ratio between the target protein and β-actin within the same sample.

### Nuclear and cytosolic fractionation

Nuclear and cytoplasmic proteins were extracted using a nuclear and cytoplasmic protein extraction kit (Beyotime, Cat. P0027) following the manufacturer’s instructions. Protein extracts were separated *via* SDS-PAGE and transferred onto a PVDF membrane. Membranes were incubated overnight at 4 °C with primary antibodies: PKM2 (Proteintech, Cat# 60268-1-Ig; diluted 1:5000), p-STAT3 (Abclonal, Cat# AP0705; diluted 1:500), STAT3 (Abclonal, Cat# A19566; diluted 1:500), β-actin (Abclonal, Cat. #AC004; diluted 1:5000), and Histone3 (Abcam, Cat# ab1791; diluted 1:1000).

### Co-immunoprecipitation

BMMs (3 × 10^6^ cells) were seeded into a 10 cm culture dish and subjected to co-immunoprecipitation (Co-IP) using the Pierce Co-Immunoprecipitation kit (Thermo Fisher Scientific) according to the manufacturer's instructions. Samples were then subjected to Western blotting.

### Mouse model of periodontitis

Experimental periodontitis was induced by the ligation method following a protocol previously described. Briefly, mice were anesthetized with an intraperitoneal injection of ketamine and xylazine, and a 5–0 silk ligature tied around the right maxillary second M by the same experienced operator (43). TEPP-46 (purity > 99%) in powder form was procured from MCE. Mice received either the target reagent (20 mg/kg of TEPP-46 every other day) or vehicle for a period of 10 days. Twenty-four animals were randomly allocated to four groups with six mice in each group: a) the non-ligated group, b) the non-ligated group receiving TEPP-46 (20 mg/kg), c) the ligated group, d) the ligated group receiving TEPP-46 (20 mg/kg). The ligated group (group c) was used as a positive control group and the non-ligated group (group a) was the negative control group. The mice in the control group also received intraperitoneal injections of DMSO without TEPP-46. After 12 days, the animals were euthanized by an intraperitoneal ketamine and xylazine overdose, and maxillae was collected.

### Bone morphology analysis

Mice were euthanized, and their right maxillary molars were dissected and fixed in 4% paraformaldehyde for 48 h. Afterward, the samples were rinsed overnight and prepared for micro-CT scanning. High-resolution micro-CT images were obtained using a Skyscan 1172 micro-CT scanner (Bruker micro-CT) operating at a 1.0 mm-thick aluminum filter. Image reconstruction was performed using the SkyScan NRecon program, and 3D reconstruction images were generated using CTvox and SkyScan CT-analyzer software, with the images adjusted to a standardized angle. To ensure consistent measurement results and avoid errors caused by inconsistent ligature ends, the region of interest for evaluating the extent of bone loss was defined. This region was delineated by a line connecting the mesial root margin of the first buccal molar, the upper boundary of the buccal alveolar bone, the root margin of the third molar, and the neck of the tooth. The micro-architectural properties of the maxillary specimens were evaluated by assessing the bone surface/volume ratio (BS/BV), bone volume fraction (BV/TV), trabecular thickness (Tb.Th), trabecular number (Tb.N), and trabecular separation (Tb.Sp).

### Hematoxylin and eosin (H&E) and tartrate-resistant acid phosphatase (TRAP) staining

The right maxillary molars of the mice were fixed in a 4% formaldehyde solution and then decalcified in a 10% ethylenediamine tetraacetic acid solution in PBS for 40 days. Following decalcification, the samples were dehydrated using a graded ethanol series, infiltrated with paraffin and sectioned at a thickness of 4 μm along the sagittal plane. The ends of the ligature are usually placed on the inner side of the mesial root of the upper second M to ensure accurate measurements and to prevent errors resulting from inconsistent ligature placement. As a result, the assessment focused on attachment loss in the distal roots of the periodontal ligament of the upper second M. Sections were stained with H&E to examine attachment loss in the distal roots of the upper second M's periodontal ligament (PDL). The extent of attachment loss (μm) was determined by measuring the distance between the cementoenamel junction and the most coronal point of connective tissue attachment. TRAP staining was performed using a leukocyte acid phosphatase kit (Servicebio, Cat.#G1050, Wuhan, China) to detect osteoclasts. The region of interest (ROI) was selected as the location of the alveolar bone surrounding the root furcation of the maxillary second M. Multinucleated (≥3 nuclei) TRAP-positive cells were identified as osteoclasts and counted.

### Immunofluorescence staining (confocal microscopy)

Cells were fixed, permeabilized, and blocked before incubation with primary antibodies. After washing, cells were incubated with fluorescently labeled secondary antibodies. Nuclei were counterstained with DAPI. Images were captured using a confocal microscope and analyzed using ImageJ software.

### Statistical analysis

All statistical analyses were conducted using GraphPad Prism V.9.0 (GraphPad Software). Data are presented as mean ± standard deviation (SD). Mean differences between the two groups were assessed using an unpaired two-tailed Student’s *t* test. For analyses involving more than two groups, either analysis of variance with multiple comparisons tests (ANOVA; for parametric data) or the Kruskal–Wallis test (for nonparametric data) was performed, followed by Bonferroni’s multiple comparisons test. A significance threshold of *p* < 0.05 was considered statistically significant.

## Data availability

Data will be made available on request.

## Supporting information

This article contains supporting information.

## Conflict of interest

The authors declare that they have no conflicts of interest with the contents of this article.
