# Supplementary material for: The glycolytic enzyme PKM2 regulates inflammatory osteoclastogenesis by modulating STAT3 phosphorylation
Source: J Biol Chem. 2025 Mar 6;301(4):108389. doi: 10.1016/j.jbc.2025.108389 (PMC11999595; doi:10.1016/j.jbc.2025.108389)
Supplement: Supplementary Table 1 [file mmc1.docx]

**SUPPLEMENTARY TABLES**

Supplementary Table 1. Genes and primer sequences for qRT-PCR

| Gene |  | Primer sequence |
| --- | --- | --- |
| *Atp6v0d2* | Forward | CATTCCTTGGAGCCCCTGAG |
|  | Reverse | TCTCTGTGAAACGGCCCAGT |
| *Dc-stamp* | Forward | TGTATCGGCTCATCTCCTCCAT |
|  | Reverse | GACTCCTTGGGTTCCTTGCTT |
| *Oscar* | Forward | GTCCGTTGAGCTGGCTGAGT |
|  | Reverse | TCTGGGGAGCTGATCCGTTA |
| *Nfatc1* | Forward | CATGCGAGCCATCATCGA |
|  | Reverse | TGGGATGTGAACTCGGAAGAC |
| *Trap* | Forward | GACAAGAGGTTCCAGGAGACC |
|  | Reverse | GGGCTGGGGAAGTTCCAG |
| *Ctsk* | Forward | ACAGCAGGATGTGGGTGTTCA |
|  | Reverse | GCCGAGAGATTTCATCCACCT |
| *Rank* | Forward | TGGGCTTCTTCTCAGATGTCTTT |
|  | Reverse | TGCAGTTGGTCCAAGGTTTG |
| *Oc-stamp* | Forward | ATGAGGACCATCAGGGCAGCCACG |
|  | Reverse | GGAGAAGCTGGGTCAGTAGTTCGT |
| *Mmp9* | Forward | CAAAGACCTGAAAACCTCCAA |
|  | Reverse | GGTACAAGTATGCCTCTGCCA |
| β-actin | Forward | CGCAGCCACTGTCGAGTC |
|  | Reverse | GTCATCCATGGCGAACTGGT |
